# Preservation of epithelial progenitor cells from collagenase-digested oral mucosa during *ex vivo* cultivation

**DOI:** 10.1038/srep36266

**Published:** 2016-11-08

**Authors:** Yi-Jen Hsueh, Shiang-Fu Huang, Jui-Yang Lai, Shih-Chieh Ma, Hung-Chi Chen, Sung-En Wu, Tze-Kai Wang, Chi-Chin Sun, Kevin Sheng-Kai Ma, Jan-Kan Chen, Chyong-Huey Lai, David Hui-Kang Ma

**Affiliations:** 1 Limbal Stem Cell Laboratory, Department of Ophthalmology, Chang Gung Memorial Hospital, Linkou, Taiwan; 2 Center for Tissue Engineering, Chang Gung Memorial Hospital, Linkou, Taiwan; 3 Department of Otolaryngology, Chang Gung Memorial Hospital, Linkou, Taiwan; 4 Department of Medicine, College of Medicine, Chang Gung University, Taoyuan, Taiwan; 5 Institute of Biochemical and Biomedical Engineering, Chang Gung University, Taoyuan, Taiwan; 6 Biomedical Engineering Research Center, Chang Gung University, Taoyuan, Taiwan; 7 Department of Ophthalmology, Chang Gung Memorial Hospital, Keelung, Taiwan; 8 Department of Chinese Medicine, College of Medicine, Chang Gung University, Taoyuan, Taiwan; 9 Department of Dentistry, College of Oral Medicine, Chung Shan Medical University, Taichung, Taiwan; 10 Department of Physiology, College of Medicine, Chang Gung University, Taoyuan, Taiwan; 11 Department of Obstetrics and Gynecology, Chang Gung Memorial Hospital, Linkou, Taiwan

## Abstract

To avoid xenogeneic infection, we report a novel protocol for producing animal-derived component-free oral mucosal epithelial cells (OMECs) sheet for transplantation, in which collagenase was used to replace dispase II/trypsin-EDTA for digesting oral mucosal tissue, and human platelet-derived PLTMax to replace fetal bovine serum. The resulting epithelial aggregates were expanded on de-epithelialized amniotic membranes without 3T3 feeder cells, and serum-free EpiLife was used to reduce contamination by submucosal mesenchymal cells. The OMEC sheets thus generated showed similar positive keratin 3/76-positive and keratin 8-negative staining patterns compared with those generated by the original protocol. Colony formation efficiency assay, BrdU label retention assay, and p63 and p75^NTR^ immunostaining results indicated that higher proliferative potentials and more progenitor cells were preserved by the modified protocol. TaqMan array analysis revealed that the transcription of integrin-linked kinase (ILK) was up-regulated along with an increase in β-catenin signaling and its downstream cell cycle modulators, cyclin D1 and p27^KIP1^. Furthermore, ILK silencing led to the inhibition of nuclear β-catenin accumulation, suppressed p63 expression, and reduced the expression of cyclin D1 and p27^KIP1^; these observations suggest that ILK/β-catenin pathway may be involved in cell proliferation regulation during the *ex vivo* expansion of OMECs for transplantation purposes.

Compared with other non-keratinized epithelia over moist mucosal surfaces of the body (e.g., oral mucosa, esophagus, vagina, and ocular surface), the corneal epithelium is highly similar to the oral mucosa. Both epithelia are stratified, with tight junction proteins, such as connexin 43 (Cx43), in the suprabasal layer, and hemidesmosome proteins, such as integrins, in the basal layer. Moreover, keratin 3/76 (detected by AE5 monoclonal antibody) is expressed in non-keratinized and stratified epithelia, including both the corneal and oral mucosal epithelia^1^; in contrast, keratin 8 is expressed in both corneal and conjunctival epithelia but is not found in oral mucosal epithelium^2^. Due to the resemblance of the two epithelia, cultivated oral mucosal epithelial transplantation (COMET), a cell therapy procedure, has been used to repair damaged corneal surfaces and as an important bridge therapy for acute or chronic corneal burns^3^. Recently, the COMET procedure has also been applied to repair intraoral mucosal defects^4^ and esophageal mucosa during endoscopic mucosal resection procedures^5^, suggesting that it has the potential for a wide variety of clinical applications.

The original protocol for the *ex vivo* cultivation of oral mucosal epithelial cells (OMECs) for COMET was first published in 2004^6^,^7^. Typically, dispase II/trypsin is used to isolate OMECs from tissues and disrupt the epithelium. To cultivate these disrupted OMECs *ex vivo*, fetal bovine serum (FBS) and 3T3 mouse fibroblasts are prerequisites as they facilitate cell adhesion and proliferation into a confluent epithelial cell sheet. From 2006 to 2009, we completed a phase I clinical trial using this protocol (approved by the Ministry of Health and Welfare of Taiwan, No. 0950206914)^3^. The trial verified the potency of COMET for promoting wound healing in severe ocular surface burns and demonstrated the long-term persistence of OMECs in the transplanted corneas^8^. Coculture with growth-arrested 3T3 cells has been a gold standard to maintain holoclone population of keratinocytes *in vitro*, and long-term success of cultivated skin or corneal epithelial cell transplantation have been well-documented^9^,^10^. In 2015, the European Commission for the first time granted a marketing authorization to Holoclar^®^ manufactured by Holostem Advanced Therapies (Modena, Italy). This autologous limbal epithelial stem cell culture product is based on the original work of Pellegrini *et al.* in which the irradiated 3T3-J2 feeder cells act through cell-to-cell interaction and paracrine effect to maintain the stemness of cultivated keratinocytes^11^,^12^,^13^. These feeder cells from certified cell bank have passed a series of biological and quality tests so that the risk of microbial or viral contamination has been minimized. However, GMP grade FBS and mouse 3T3 cells are difficult to procure. Moreover, factors containing undefined serum contents are not ideal for standardizing culture protocols^14^,^15^. Therefore, we endeavored to develop an animal-derived component-free (ADCF) culture procedure.

Several different cell carriers have been developed to fabricate epithelial cell sheets for COMET, including thermoresponsive interfaces^7^, fibrin^16^, and denuded amniotic membrane (AM)^6^. More recently, Hyun *et al.* reported to generate biomaterial-free OMEC sheets using collagenase/trypsin digestion and coculture with 3T3 cells^17^. Denuded AM has been used for ocular surface reconstruction surgery for more than two decades with satisfactory results^18^,^19^. AM effectively preserve epithelial stem cells when used as a carrier for cultivating limbal epithelial cells^20^,^21^, and evidence has shown that OMECs cultivated on AM still exist almost two years after transplantation^8^. In addition, AM has been shown to effectively inhibit inflammatory reactions during ocular surface wound healing^19^. Accordingly, we continued to use denuded AM as a cell carrier in our modified protocol. In 2011, Chen *et al.* reported the use of collagenase to replace dispase II/trypsin to digest corneal limbal tissues (containing corneal epithelial stem cells) and generate epithelial cell aggregates. Such aggregates, which contain epithelial basement membrane (EBM) proteins and sub-EBM mesenchymal cells, preserved stem/progenitor cell characteristics^22^ and improved their proliferative potentials^23^,^24^. Therefore, in this study, we attempted to isolate OMECs with collagenase and generate epithelial sheets in the absence of 3T3 feeder layers.

When epithelial cells are isolated by dispase II/trypsin, the EBM is degraded; but when the cells are isolated by collagenase, the EBM can be maintained. Consequently, we speculate that when cell aggregates are generated after collagenase treatment, cellular proliferation may be regulated through cell-EBM interactions. Cell-EBM interactions transfer intracellular signals through EBM receptors (i.e., integrins), resulting in enhanced cell proliferation^25^,^26^. Moreover, integrin signaling mediates the cell cycle through the PI3K/ERK pathway^27^, which interferes with cyclin D1 via the ILK/GSK3 pathway^28^,^29^ and modulates cell proliferation by inhibiting p27^KIP1^ through the FAK/Rho/ROCK pathway^30^,^31^.

In this study, we successfully refined the fabrication process for OMEC sheets using ADCF products after collagenase-facilitated cell isolation. Importantly, we found that this refined protocol yielded cell sheets with increased proliferative potential. Furthermore, we demonstrated that ILK/β-catenin pathway activation is involved in this enhanced cell proliferation.

## Results

**Figure 1:**
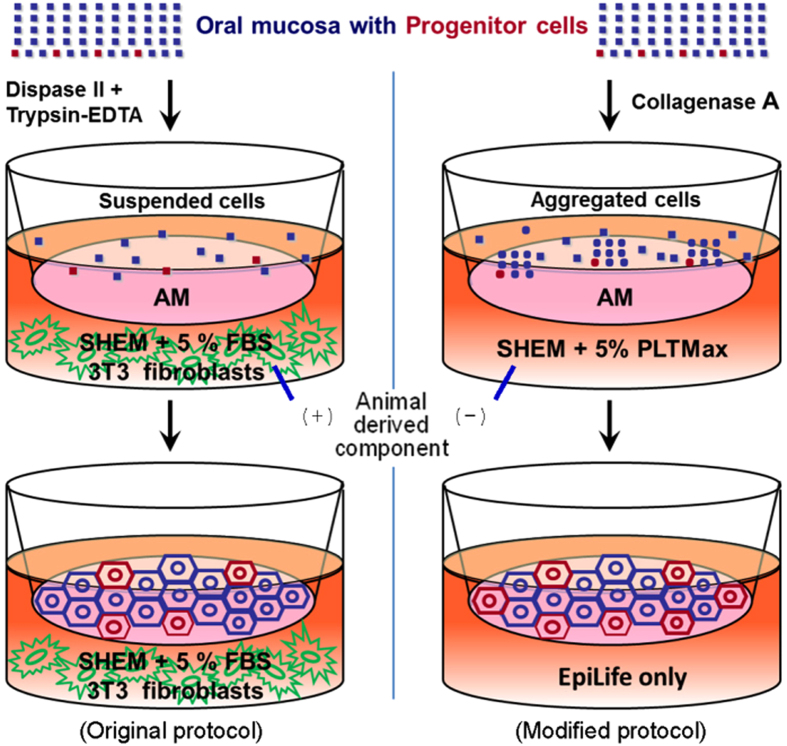
Comparison of original and modified protocols for the *ex vivo* cultivation of human oral mucosal epithelial cells (OMECs). Left panel: In the original protocol, oral epithelium was isolated from oral mucosa with dispase II. Suspended OMECs were then dissociated with trypsin-EDTA, re-suspended and cultivated on de-epithelialized amniotic membrane (AM) with 3T3 fibroblasts as a feeder layer, in SHEM containing fetal bovine serum (FBS). Right panel: In the modified protocol, oral mucosal tissues were treated with collagenase A. The resulting cell aggregates were grown on AM without a 3T3 feeder layer. In addition, FBS was replaced with PLTMax. Two days after seeding, the culture medium was changed to serum-free medium (EpiLife) to prevent the overgrowth of submucosal fibroblasts.

To prevent concerns about uncontrolled components and zoonosis from FBS and 3T3 fibroblasts, we developed a novel ADCF culture procedure for OMEC sheet preparation (Fig. 1) to improve the safety and function of this cell culture product for clinical applications.

### Collagenase-isolated OMECs formed cell sheets in the absence of 3T3 feeder layers

**Figure 2:**
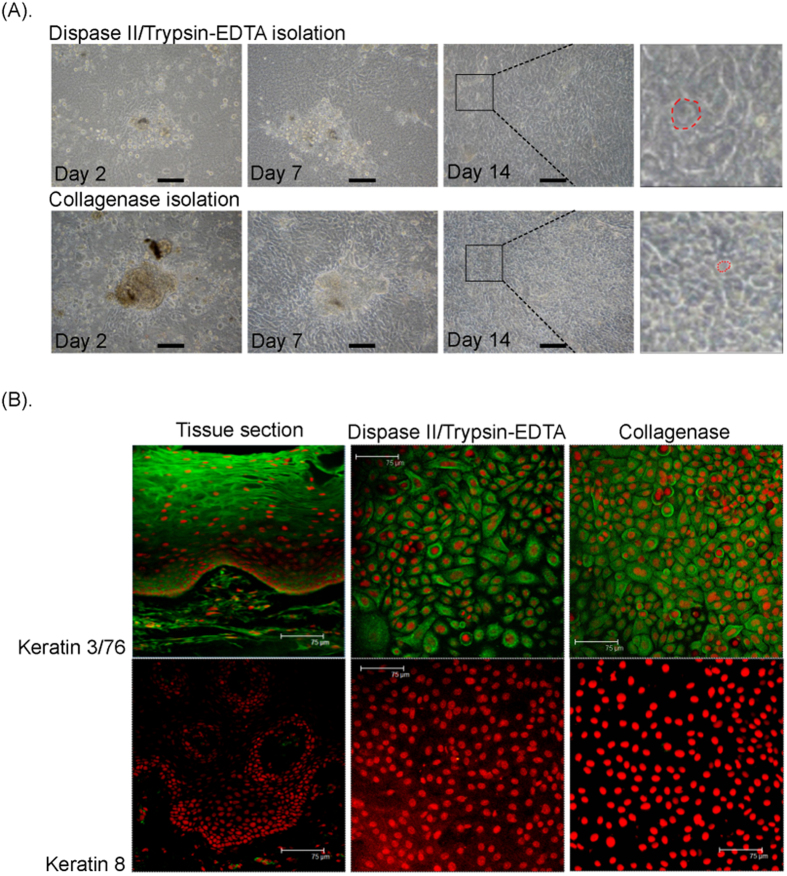
Phase contrast microscopy and keratin immunostaining for human OMECs isolated by collagenase or dispase II/trypsin-EDTA. (**A**) Phase contrast microscopy for human OMECs seeded on AMs after isolation. Close-up views of selected areas in the photo of cells at Day 14 are indicated by square brackets, whereas approximate cell size is indicated by a dotted red line. Human OMECs isolated by collagenase exhibited a more compact morphology. The bars represent 100 μm. (**B**) In oral mucosal tissue, cytokeratin 3/76 (K3/76; green) was expressed in non-keratinized epithelia, whereas cytokeratin 8 (K8; green) was not expressed. When human OMECs were cultivated on the AM surface for 14 days, similar K3/76-positive and K8-negative staining patterns were exhibited in both the collagenase (Col.) or dispase II/trypsin (D/T) treated groups. The bars represent 75 μm.

After an overnight collagenase digestion, OMEC aggregates adhered to the denuded AM surface, which was aided by adding the FBS substitute PLTMax to supplemented hormonal epithelial medium (SHEM). Two days after cell adhesion, we replaced the SHEM with EpiLife medium, which is a chemically defined serum-free medium; this step was performed to promote cell aggregate expansion and proliferation without the use of 3T3 feeder layers and to prevent contamination from submucosal mesenchymal cells (Fig. S1). Fourteen days after seeding, the primary culture of OMECs reached confluency on denuded AM, and we observed an OMEC morphology more compact than that generated by the original protocol (Fig. 2A), in which confluency usually took 3 weeks. Previously, we have shown that cytokeratin 3/76 (K3/76) was expressed in non-keratinized corneal, conjunctival, and oral mucosal epithelia, whereas cytokeratin 8 (K8) was expressed in corneal and conjunctival epithelia, but not in oral mucosal epithelia^8^. Similar to OMECs cultivated according to the original protocol (i.e., the dispase II/trypsin treatment procedure), the cells cultivated according to our modified protocol were also K3/76 positive and K8 negative after cultivation on denuded AM for 14 days (Fig. 2B). The expression of Cx43 and cytokeratin 13 (K13) in culture was similar to that in oral mucosal epithelium (Cx43+and K13+) (Fig. S2).

### Modified protocol preserves higher proliferative potential of OMECs

**Figure 3:**
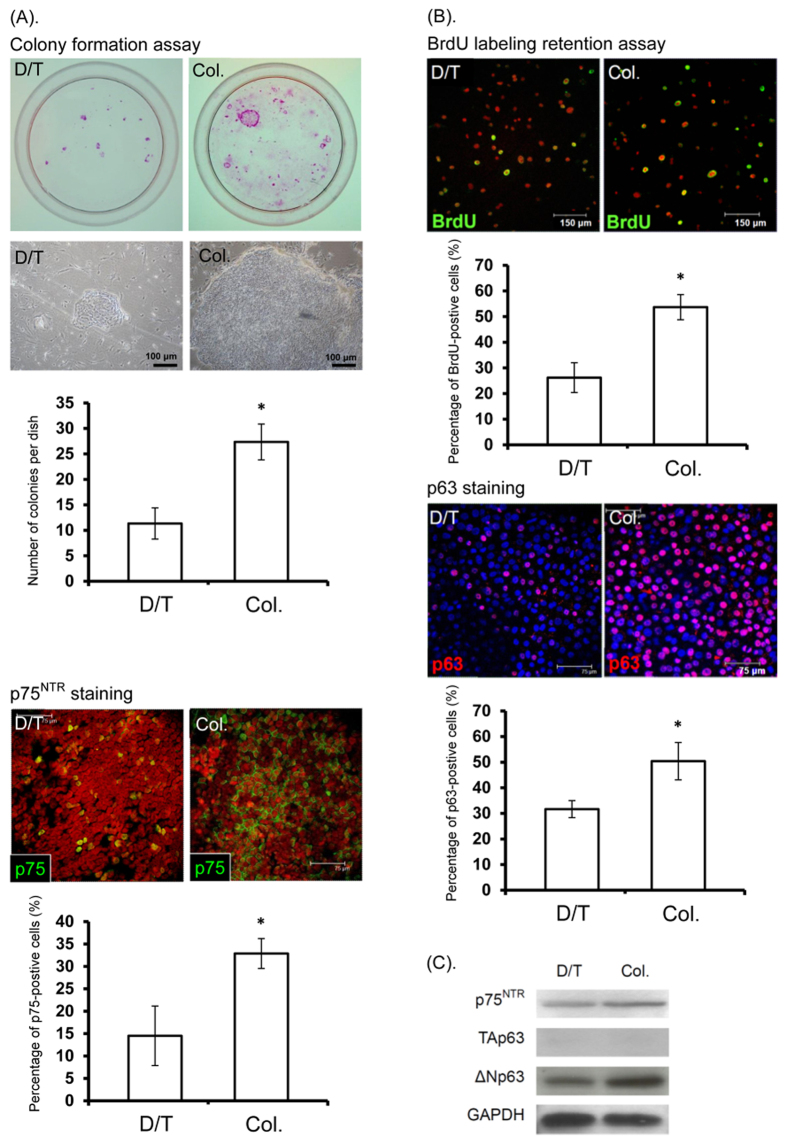
Colony formation assay (**A**) and BrdU label retention assay (**B**) for human OMECs isolated by dispase II/trypsin-EDTA (D/T) or collagenase (Col). (**A**) More colonies (first row, **A**) and larger colonies (second row, A) were formed from OMECs isolated by collagenase, indicating a higher proportion of progenitor cells (*p < 0.05). Moreover, higher p75^NTR^ expression was observed in the collagenase-isolated group (p75, green; bottom row, A; *p < 0.05). (**B**) Retained BrdU labeling (green) after additional 2 weeks of culture was significantly increased in the collagenase-isolated group (first and second rows, (**B**); *p < 0.05). Moreover, higher p63 expression (red) was observed in the collagenase-isolated group (third row, B; nuclei were counterstained with Hoechst 33342; *p < 0.05). (**C**) Western blotting for p75^NTR^, TAp63, and ΔNp63 expressed by OMECs isolated by dispase II/trypsin-EDTA or by collagenase.

More colonies formed from OMECs cultivated according to the modified protocol (27.3 ± 3.5 vs 11.3 ± 3.1 colonies, *p* = 0.049, n = 3), which suggests a better proliferative potential. Moreover, larger colonies were generated with the modified protocol, indicating that progenitor cells were better preserved with the modified protocol (Fig. 3A). In addition, there was a higher proportion of p75^NTR^-positive cells in the experimental cultures (32.9 ± 3.3% vs 14.5 ± 6.7%, *p* = 0.009, n = 5). As p75^NTR^ is primarily expressed in the basal layers of the oral mucosal epithelium, the increase in p75^NTR^-positive cells may represent a pro-progenitor characteristic of the cultured OMECs^32^. In contrast with the sparse distribution of p75^NTR^-positive cells in OMECs cultivated according to the original protocol, we observed a clustering of p75^NTR^-positive cells in OMECs cultivated according to the modified protocol, suggesting that p75^NTR^-positive cells were maintained in cell sheets that expanded from cell aggregates (Fig. 3A).

We next performed a BrdU labeling assay to examine cell proliferation. Immunostaining showed that the percentage of BrdU-labeled (53.7 ± 4.9% vs 26.2 ± 5.8%, *p* = 0.009, n = 5) and p63-positive cells (50.4 ± 7.3% vs 34.7 ± 3.3%, *p* = 0.009, n = 5) was significantly increased in the collagenase-treated group (Fig. 3B). Immunoblotting showed that the ΔNp63 but not the TAp63 isoform of the transcription factor p63 was predominantly expressed in OMECs cultivated *ex vivo* (Fig. 3C). The ΔNp63 isoform was previously regarded as a stem cell marker in epithelial tissue^33^, but more recent reports have shown increased ΔNp63 expression in wound healing and epithelial cells cultivated *ex vivo*^34^,^35^. Taken together, these results suggest that ΔNp63 expression may reflect cellular proliferative status in cultures.

### Increase in ILK pathway-related gene expression in collagenase-isolated OMECs cultivated *ex vivo*

As the collagenase isolation procedure does not perturb extracellular structures (e.g., the EBM and extracellular domains of transmembrane proteins), the interactions between integrins and their ligands are maintained in collagenase-treated OMEC aggregates. Accordingly, we performed a 96-well plate ILK signal assay to determine whether ILK-related gene expression was up-regulated in OMECs isolated by the modified protocol. The results demonstrated that the expression of genes related to cell membrane structures was significantly higher in OMECs isolated with the modified protocol, including β2 integrin, E-cadherin, and EGFR. Integrin pathway-related genes (e.g., ILK and AKT), β-catenin pathway-related genes (e.g., GSK-3β and TCF4), and down-stream proliferation/migration-related genes (e.g., Rho/Rac, c-Jun, and cyclin D1) were also highly expressed (Table 1). We attribute these findings to the positive feedback regulation of integrin-ligand interactions^36^. ILK plate array was performed as a screening tool in gene expression, though performed only once, the trends in upregulated pathways of ILK, WNT, and cell proliferation was later verified by Western blot.

### Increase in ILK/β-catenin pathway activity in *ex vivo* collagenase-isolated OMECs

**Figure 4:**
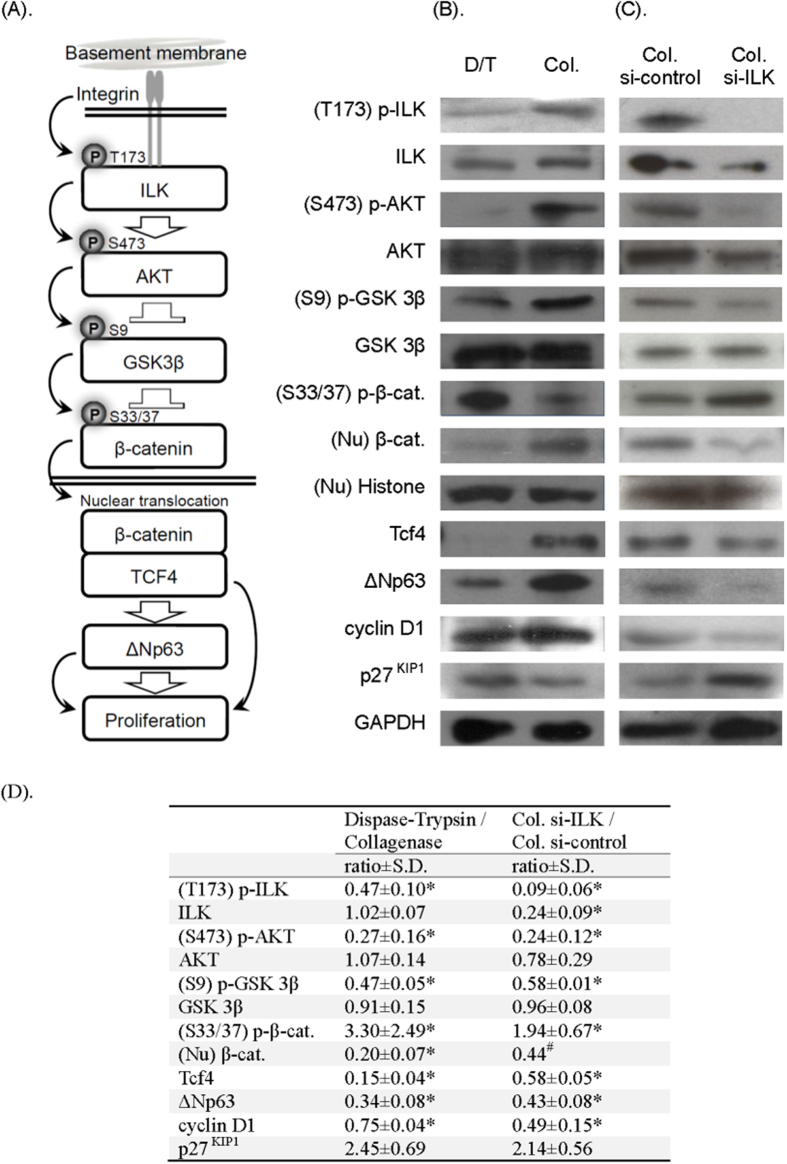
ILK/β-catenin/p63 signaling cascade expressed by human OMECs isolated by dispase II/trypsin-EDTA (D/T) or collagenase (Col). (**A**,**B**) Upon OMEC attachment to the basement membrane via integrin receptors, ILK is phosphorylated and activated. This not only phosphorylates and activates AKT but also phosphorylates and inactivates GSK-3β, such that β-catenin can avoid degradation and translocate to the nucleus. In the nucleus, β-catenin works with other transcription factors, such as TCF4, to turn on Wnt-associated genes, such as ΔNp63 and cyclin D1, to promote cell proliferation (activated ΔNp63 down-regulates p27^KIP1^). (**C**) Effect of ILK silencing on the signaling pathway. The causal relationship of the ILK/β-catenin/p63 pathway was confirmed by transfecting ILK siRNA into OMECs isolated by collagenase. Consequently, when the ILK pathway was blocked, nuclear β-catenin translocation, p63 expression, and cell cycle mediators were all significantly suppressed. (**D**) The immunoreactive band density of each experiment was quantified, first by normalization by the density of internal control (GAPDH or histone H3), then the relative signal intensity was expressed as D/T group divided by Col. group (with or without ILK silencing). Data are expressed as mean ± SD from three independent experiments. *p < 0.05. ^#^Two test results with samples pooled from three donors.

We have previously shown that extracellular matrix surface geometry influences ILK pathway activity in epithelial cells, and activates the β-catenin pathway by inhibiting GSK-3β activity^36^. ILK activation increases its kinase activity and suppresses GSK-3β activity by phosphorylating site S9. Suppressed GSK-3β decreases β-catenin degradation through the down-regulation of β-catenin phosphorylation at site S33/37, resulting in the nuclear translocation of β-catenin. We have also previously reported that the promoter region of the transcription factor ΔNp63 contains β-catenin binding sites; therefore, the activation of Wnt signaling may turn on ΔNp63, the master regulator of keratinocyte stem cells^37^. In epithelial cells, the β-catenin pathway can promote cell proliferation directly, through increased cyclin D1 expression^38^, or indirectly, via suppression of the CDK inhibitor p27^KIP1^ through p63 pathway activation^39^,^40^ (Fig. 4A).

In order to determine whether ILK/β-catenin pathway activation is different in OMECs isolated with collagenase or dispase II/trypsin, we performed immunoblotting to analyze the phosphorylation of signal pathway-related molecules and the nuclear translocation of β-catenin. We found that in the collagenase-isolated group, the expression of phospho-ILK(T173), phospho-AKT(S473), and phospho-GSK 3β(S9) and the nuclear translocation of β-catenin were up-regulated and that the expression of phospho-β-catenin(S33/37) was down-regulated (Fig. 4B). However, the expression of phospho-AKT (T34), phospho-GSK 3β (Y216), and phospho-β-catenin (S552) remained unchanged (data not shown). Moreover, we found increased expression of TCF4, ΔNp63, and cyclinD1, whereas the expression of p27^KIP1^ was decreased, indicating increased ILK/β-catenin pathway activity and up-regulated expression of cell cycle modulators in the collagenase-isolated group.

### ILK signaling modulates proliferation control of collagenase-isolated OMECs cultivated *ex vivo*

The expression of ILK mRNA in OMECs was silenced to determine the effects of ILK signaling on the β-catenin pathway and proliferation control in the collagenase-isolated OMECs. After inhibiting ILK transcription, nuclear β-catenin translocation and ΔNp63 and cyclin D1 expression were significantly reduced, whereas p27^KIP1^ expression increased (Fig. 4C), suggesting a critical role for ILK signaling in the proliferation control of collagenase-isolated OMECs.

### Presence and distribution of submucosal mesenchymal cells in collagenase-isolated OMECs

**Figure 5:**
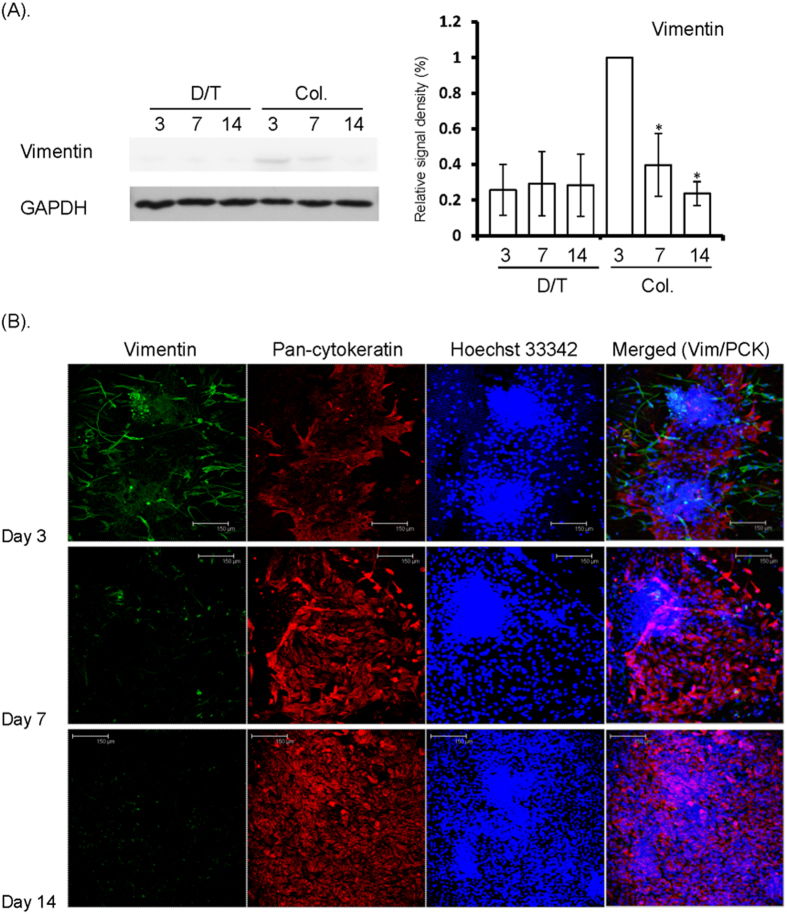
(**A**) Western blot for vimentin expression by OMECs isolated by dispase II/trypsin or collagenase. Vimentin expression was evident in Day 3 collagenase-isolated cultures, implying the presence of submucosal mesenchymal cells. Under serum-free culture conditions, the signal for vimentin dramatically diminished in Day 7 cultures and was absent in Day 14 cultures. On the contrary, the signal for vimentin was persistently negative in dispase II/trypsin-EDTA-isolated cultures. The immunoreactive band density of each experiment was quantified by densitometric analysis, followed by normalization to the level of internal controls (GAPDH), and was expressed as a ratio to the highest density in Day 3 Col. group. Data are expressed as mean ± SD from three independent experiments. *Value significantly lower than that of Day 3 (*p* < 0.05). (**B**) Whole-mount immunoconfocal microscopy for vimentin and pan-cytokeratin expressed by OMECs isolated by collagenase. In Day 3 cultures, immunostaining for vimentin (Vim, green) was prominent in collagenase-isolated OMEC aggregates (positive for pan-cytokeratin; PCK, red), indicating the presence of mesenchymal cells. Staining for vimentin decreased dramatically by Day 7 of serum-free culture and was essentially absent by Day 14. Cell nuclei were counterstained with Hoechst 33342 (blue).

In this study, early trials of cell sheet cultivation by collagenase isolation revealed serious contamination by fibroblasts (submucosal mesenchymal cells), which did not occur in the dispase II/trypsin-isolated group. We reasoned that the contamination was related to the serum component in the culture medium; therefore, we later used serum-free EpiLife medium instead. As the immunoblotting results show in Fig. 5A, the expression of vimentin, a mesenchymal cell marker, was barely detectable in the dispase II/trypsin-isolated group, whereas vimentin expression decreased over time in the collagenase-isolated group. These data suggest that the growth of submucosal mesenchymal cells can be suppressed in serum-free medium (e.g., EpiLife). We next performed whole-mount immunostaining to monitor the distribution of mesenchymal cells (vimentin-positive) and OMECs (pan-cytokeratin-positive) during the cultivation period (Fig. 5B). One day after the switch to EpiLife medium (Day 3), numerous fusiform mesenchymal cells were observed around the cell aggregates. As the time in serum-free medium increased, the number of keratinocytes increased, whereas the number of mesenchymal cells decreased. Over time, the mesenchymal cells decreased in number and size, and lost the ability to proliferate. In addition, and importantly, vimentin-positive cells were no longer present in the confluent culture.

In conclusion, collagenase isolation of OMECs followed by serum-free culture allows the generation of cultivated cell sheets without the need for animal-derived products and is free from mesenchymal cell contamination. The OMEC sheets generated by using collagenase isolation contained more progenitor cells and demonstrated increased proliferation potential compared with those generated by using dispase II/trypsin isolation. The preservation of OMEC EBM components after collagenase treatment activates the integrin-related pathways, which in turn activates the ILK and AKT signaling pathways. ILK then phosphorylates and inactivates GSK-3β, thereby preventing the degradation and facilitating the nuclear translocation of β-catenin, resulting in increased cell proliferation.

## Discussion

In addition to applications in ocular surface reconstruction, tissue-engineered OMEC sheets have recently been validated for repairing oral mucosal defects after dental surgery^4^ and for repairing esophageal mucosa after endoscopic mucosal resection^5^, indicating that this technology has clinical importance and wide applicability. However, to meet the increasingly strict regulations on cell therapies, we have extensively investigated cultivation protocols using reagents free of animal sources. By using ADCF product, various screening tests on xenogeneic diseases can be omitted, and cell culture procedure can become simpler.

We initially attempted to replace FBS with autologous serum. However, neither the time required for cultivation nor the quality of cell products was consistent due to variation in the efficacy of autologous serum among individuals. As good manufacturing practices-certified cell culture reagents have been developed to comply with the increasingly strict standards for cell therapy products, we chose to substitute FBS with PLTMax, a commercially available product derived from human platelet lysate. The addition of PLTMax promotes cell adhesion after isolation because OMEC aggregates attach poorly in the absence of serum.

Recent evidence has shown that corneal epithelial stem cells are regulated by mesenchymal cells located in the stroma of the limbus, where epithelial stem cells reside^41^,^42^,^43^. Previously, human mesenchymal stem cells^44^ and human dermal fibroblasts^45^ have been evaluated as a substitute feeder cell layer to cultivate human limbal and oral mucosal epithelial cells respectively with some success. Earlier, we replaced 3T3 mouse feeder layers with limbal stromal mesenchymal cells; however, they were unsuccessful in supporting OMEC proliferation. Recently, the isolation of epithelial cells by collagenase has been suggested to maintain basement membrane components and increase cell culture efficiency. Therefore, we adopted this method to establish a cell culture protocol without the need for a feeder cell layer. The collagenase isolation technique generated epithelial aggregates that expanded into confluent epithelial sheets, but with frequent contamination by submucosal mesenchymal cells. We overcame this problem by replacing the medium with a chemically defined serum-free medium (i.e., EpiLife) to inhibit mesenchymal cell proliferation. In this study, we observed a higher CFE and BrdU label retaining ability in collagenase-treated OMECs. However, we cannot readily assume that the progenitor cell-preserving capability can be superior in the absence of feeder layer, because the isolating enzymes and supplemented sera in the two methods were different, and in our study the feeder layer was separated from OMECs by a culture insert, therefore only soluble factors secreted by 3T3 cells can reach OMECs. Additionally, direct contact of OMECs with 3T3 cells may benefit from the growth-promoting extracellular matrix proteins secreted by the latter, which we did not study.

Earlier studies have suggested that sub-EBM mesenchymal cells not only are involved in modulating epithelial cell proliferation and differentiation^46^ but also modulate the β-catenin pathway in the corneal epithelium through DKK1/2^47^. In collagenase-treated cultures, although submucosal mesenchymal cells were initially present in the cell aggregates, the proportion of submucosal mesenchymal cells diminished when cultured under serum-free conditions, rendering OMECs the predominant cell type (Fig. 5). Therefore, we speculate that in the early phase, submucosal mesenchymal cells might function as the 3T3 cells do to enhance the attachment and proliferation of OMECs; however, this role of mesenchymal cells or potential niche cells remains to be elucidated.

Immunoblotting analysis of the ILK/β-catenin pathway revealed discrepancies in TCF4 and cyclin D1 expression between the dispase II/trypsin- and collagenase-isolated groups. We observed alterations in key factors, such as AKT phosphorylation and β-catenin nuclear translocation; therefore, we intended to examine the role of the ILK pathway in the collagenase-isolated group. We used siRNA to silence ILK expression in an effort to monitor changes in the β-catenin pathway and its downstream effectors. The immunoblotting results demonstrated diminished β-catenin nuclear translocation and profound changes in its downstream factors, e.g., p63, cyclin D1, and p27^KIP1^, suggesting a dominant role of the ILK pathway in regulating proliferation in the collagenase-isolated group.

Previously, we found that p63 primarily exerts its effects on epithelial cell proliferation through p27^KIP1^ but only mildly through cyclin D1^39^,^40^. In contrast, cyclin D1 has been reported to be the major downstream effecter of β-catenin/TCF in modulating epithelial cell proliferation^28^,^29^. Therefore, we hypothesize that when the ILK pathway is blocked in OMEC cultures, decreased cyclin D1 expression is a consequence of β-catenin/TCF complex suppression; whereas increased p27^KIP1^ expression results from p63 down-regulation. Nevertheless, these hypotheses require further clarification.

In this study, we continued to use AM as a cell culture substrate. AM has been used as a substrate to promote the growth of transplanted corneal epithelial stem cells^48^. Subsequent research has shown that AM transplantation has potent anti-inflammatory effects^49^ and effectively preserves corneal epithelial stem cells in culture^20^. Importantly, stem cell-preserving factors in AM have recently been isolated and identified^21^. Therefore, the ease of manipulating cultivated OMEC sheets during transplantation surgery, the preservative effects on oral mucosal epithelial progenitor cells, and the anti-inflammatory effect of AMs support the continued use of AM as a successful cell carrier. Based on observation from this study, we have established the minimal requirements for the cultures to be qualified for transplantation, i. e., the mature culture should have a cell density over 2000/mm^2^, viability of cells over 90%, positive immunostaining for p63 (using 4A4 antibody) over 40%, and over 80% for K3/76 staining (using AE5 antibody).

In summary, to avoid xenogeneic infection from animal-derived products, we developed a novel protocol for producing ADCF oral mucosal epithelial sheets for transplantation. This protocol replaces dispase II/trypsin with collagenase for digestion of oral mucosal tissue and uses serum-free culture medium to effectively eliminate contamination by submucosal mesenchymal cells. Our results indicated that OMECs isolated by the modified protocol exhibited a higher proliferative potential, and ILK/β-catenin pathway activation may be involved in the regulation of keratinocyte proliferation during *ex vivo* expansion.

## Methods

### Materials

DMEM:F12 (1:1), Opti-MEM, EpiLife medium, Supplement S7, trypsin-EDTA, phosphate-buffered saline (PBS), gentamicin, amphotericin B, Lipofectamine 2000 and Alexa-Fluor-conjugated secondary IgG were purchased from Invitrogen (Carlsbad, CA). Dimethyl sulfoxide (DMSO), methanol, and Triton X-100 were purchased from Sigma-Aldrich (St. Louis, MO). PLTMax was purchased from Mill Creek Life Sciences (Rochester, MN). Collagenase A was purchased from Roche Applied Science (Indianapolis, IN). Recombinant human EGF was purchased from Upstate Biotechnology Inc., (Lake Placid, NY). Hoechst 33342 dye was purchased from Upstate Biotech (Waltham, MA). Propidium iodide, control siRNA and ILK siRNA were purchased from Santa Cruz Biotechnology (Santa Cruz, CA). All plastic cell culture wares were obtained from Corning Incorporated Life Sciences (Acton, MA).

Rabbit polyclonal antibodies against ILK, histone H3, phospho-β-catenin (S33/37), and phospho-AKT (S473, clone 193H12), phospho-GSK-3β (S9, clone 5B3) rabbit monoclonal antibodies were purchased from Cell Signaling Technologies (Beverly, MA). Pan-cytokeratin (clone AE1/AE3) mouse monoclonal antibody was purchased from Dako (Carpinteria, CA). AKT (H-136), GSK-3β (H-76), GAPDH (FL-335), p27^KIP1^ (C-19) rabbit polyclonal, and p75^NTR^ (C20) goat polyclonal, and cytokeratin 8 (clone C51) mouse monoclonal antibodies were purchased from Santa Cruz Biotechnology (Santa Cruz, CA). Cytokeratin 13 (clone 1C7), β-catenin (clone 15B8), cyclin D1 (clone CD1.1) mouse monoclonal, and TCF4 (clone EP2033Y), vimentin (clone SP20) rabbit monoclonal antibodies were purchased from Abcam (La Jolla, CA). BrdU mouse antibody (RPN20Ab) was purchased from Amersham, GE Healthcare (Chalfont St Giles, UK). Connexin 43 (clone 4E6.2), p63 (clone 4A4) and cytokeratin 3/76 (clone AE5) mouse monoclonal antibodies were purchased from Chemicon, Millipore (Billerica, MA). Phospho-ILK (T173) rabbit polyclonal antibody was purchased from Abgent (San Diego, CA). TAp63 and ΔNp63 rabbit polyclonal antibodies were purchased from BioLegend (San Diego, CA).

### Cell culture

Human oral mucosal tissue and amniotic membrane (AM) were obtained in accordance with the tenets of the Declaration of Helsinki for research involving human subjects with approval from the institutional review board of Chang Gung Memorial Hospital in Taoyuan, Taiwan (IRB approval number 101-0108B and 102-5895B). Oral mucosal tissues were obtained from 30 patients (M: F = 26: 4, mean age = 53.8 ± 13.8 years; range: 21 to 85 years) during oral surgery. Informed consent to use the tissue for study was obtained from every donor, and these were normal tissues based on pathology report (Table S1). The AM was obtained after elective Cesarean section and tested negative for hepatitis B and C virus, human immunodeficiency virus, and syphilis. The AM was preserved in 1:1 DMEM/glycerol at −80 °C, and upon experiment, the AM was thawed, rinsed with PBS and immersed in 0.02% EDTA in PBS at 37 °C for 1 h, followed by gentle removal of the epithelium with a cell scraper (Corning). The 1.5 × 1.5 cm de-epithelialized AM was laid on a 25-mm culture insert and air-dried overnight before use.

The original protocol (i.e., dispase II/trypsin isolation) was performed as previously described^3^. In brief, the tissue was rinsed and treated with 100 μL 1.2 IU dispase II in PBS at 37 °C for 1 h. The tissue was transferred to another 35-mm dish and treated with 75 μL of 0.25% trypsin-EDTA solution at 37 °C. The cells were then collected by gently scraping the tissue surface with a cell scraper, and the trypsin solution containing the cells was collected and neutralized with SHEM containing DMEM/Ham’s F12 (1:1, 20 mM HEPES buffer) supplemented with 5% FBS, 0.5% DMSO, 2 ng/mL recombinant human EGF and 1 mg/mL recombinant insulin. Following centrifugation at 950 g, the cells were resuspended in 1.5 mL of culture medium and plated onto 25-mm transwell inserts overlaid with a layer of denuded AM. The transwell inserts were cocultured with mitomycin C-pretreated 3T3 fibroblast feeder cells (ATCC cat. no. 1658) in a six-well plate.

For the collagenase-isolated cell culture protocol, the tissue was first cut into tiny pieces, then added to 1.5 mL Eppendorf tube containing 1 mL 0.5 mg/mL collagenase A in serum-free SHEM. The tube was kept in an orbital shaker at 37 °C overnight. At the completion of incubation, the cells were spun down with an Eppendorf centrifuge at 4 °C, 3,500 rpm for 5 minutes to remove the supernatant. The cells were seeded onto denuded AM overlaid on a 25 mm transwell insert and cultured with 1.5 mL SHEM containing the same ingredients, except that 5% FBS was replaced by 5% PLTMax. Two days later, the medium was replaced by serum-free EpiLife medium (containing 1% Supplement S7). The medium was changed every 3 days until experiment, and except in colony formation efficiency assay, only the P0 primary culture cells were used for experiments.

### Gene expression assay

After one week of culture (beginning from cell seeding), the cells were harvested and RNA was extracted using TRI Reagent Solution (Ambion Inc.). cDNA synthesis was performed using High Capacity cDNA Reverse Transcription Kit (Applied Biosystems Inc.). Gene expression analysis was carried out in a TaqMan Human ILK Signaling Array (Applied Biosystems Inc.) using 5 μl TaqMan Gene Expression Master Mix (2×) (Applied Biosystems Inc.), with 5 μl cDNA in each well. PCR reactions were monitored in real time using the ABI 7900HT Fast Real-time PCR system (Applied Biosystems Inc., Foster City, CA). The thermal cycling conditions for real-time PCR were 50 °C for 2 mins, then 95 °C for 20 secs, and 40 cycles of denature (95 °C, 3 secs) and annealing/extension (60 °C, 30 secs). Relative quantitation of gene expression was determined using the ΔΔCt method.

### ILK siRNA transfection

ILK siRNA and non-targeting siRNA were purchased from Santa Cruz. OMECs were cultured to Day 7 and were incubated with Opti-MEM overnight. Then, the cells were transfected with ILK siRNA (50 nM) using Lipofectamine 2000 for 6 h. Control vector or non-targeting siRNA were used as controls for nonspecific effects. After transfection, the cells were cultured in EpiLife medium for further examination. The experiment was repeated for 3 times.

### Immunofluorescence staining

After two weeks of culture, OMECs on denuded AM were fixed in 4% formaldehyde for 15 min at room temperature, rinsed with PBS, permeabilized with 0.2% Triton X-100 for 15 min, and rinsed with PBS. After incubation for 30 min with 2% bovine serum albumin to block nonspecific staining, the cells were incubated with primary antibodies (all at 1∶100 dilution) for 24 h at 4 °C. After being washed with PBS, the cells were incubated with the corresponding Alexa-Fluor (488 or 594)-conjugated secondary IgG antibodies for 60 min at room temperature. Cell nuclei were counterstained with Hoechst 33342 or propidium iodide. Sections were mounted with Gel Mount (Biomeda, Foster City, CA) and examined using a Zeiss fluorescent microscope (Oberkochen, Germany) or a confocal microscope (Leica, Deerfield, IL). Each staining was repeated for 5 times.

### Bromodeoxyuridine (BrdU) label retention

The BrdU labeling procedure has been previously described^2^. In brief, one-week-old cultures (n = 3) were fed with DMEM with 5% FBS containing 1:500 diluted labeling reagent for 1 week, then chased for 2 weeks in BrdU-free medium. The culture was fixed with 100% pre-chilled methanol for 10 minutes. Nonspecific binding was blocked with 5% normal donkey serum (NDS) in PBS for 30 minutes, reconstituted nuclease/BrdU antibody was added and incubated for 1 hour at room temperature. Subsequently, the culture was incubated with Alexa-Fluor-conjugated secondary IgG for another 30 minutes and then was counterstained with propidium iodide (PI). The label retention percentage was calculated by dividing the number of BrdU-positive nuclei by the total number of PI-positive nuclei in five randomized fields.

### OMECs colony formation assay

NIH/3T3 cells were treated with 4 μg/mL mitomycin C at 37 °C for 2 h and seeded on 35-mm dishes at 2 × 10^4^ cells/cm^2^. Subsequently, two-week-old cultivated OMECs on denuded AM (n = 3) were treated with 1.5 U dispase II for 15 min at 37 °C, isolated by trypsin, and then seeded on dishes plated with mitomycin C-treated 3T3 feeder cells at a density of 5 × 10^2^ cells/cm^2^. The medium was changed every 3 days until Day 12, when the cells were fixed in 4% paraformaldehyde in PBS for 10 min. The cells were then stained with 2% (wt/vol) aqueous solution of rhodamine-B (Panreac, Kuurne, Belgium) for 30 min. Colony formation efficiency was calculated by dividing the number of colonies by the number of seeded OMECs.

### Protein extraction and Western blotting

Each culture dish was washed once with ice-cold PBS, and the epithelial layers were isolated by treatment with 1.5 U dispase II for 15 min at 37 °C. Isolated OMECs were suspended in 0.5 mL of Tissue Protein Extraction Reagent (T-PER; Pierce, Rockford, IL). The T-PER was supplemented with 10 mM sodium fluoride, 10 mM sodium orthovanadate, and a 1× protease inhibitor cocktail (Sigma-Aldrich). The suspension was transferred to an Eppendorf tube on ice, sonicated to break the cells, and centrifuged in a microfuge (Labnet, Edison, NJ) for 15 min at 4 °C at full speed. The supernatant was pooled and designated as total protein extract.

Nuclear proteins were extracted using a Nuclear Extraction kit (Affymetrix, Santa Clara, CA) according to the manufacturer’s instructions. In brief, cultures were washed twice with cold PBS and incubated in a 10× volume of a cytosolic extraction buffer (supplemented with 1 mM dithiothreitol, 1× protease and phosphatase inhibitor) on ice for 10 min. The nuclei were scraped, and the nuclear clump was disrupted by repetitive pipetting. The suspension was then centrifuged at 14,000 g for 3 min at 4 °C. The pellet was resuspended in 150 μL of nuclear extraction buffer supplemented with 1 mM dithiothreitol, 1× protease and phosphatase inhibitor and vortexed at full speed for 10 sec. The sample was incubated on ice for 2 h, shaken every 20 min, and centrifuged at 14,000 g for 5 min at 4 °C. The supernatant was collected and designated as the nuclear protein extract.

The concentration of protein extract was determined using a Bio-Rad protein assay kit (Bio-Rad, Hercules, CA), and then equal amounts of protein were resolved on acrylamide gradient gels and transferred to polyvinylidene difluoride (PVDF) membranes (Millipore). The membranes were blocked with 5% (w/v) fat-free milk in TBST (50 mM Tris-HCl, pH 7.5, 150 mM NaCl, 0.05% (v/v) Tween-20) and probed at 4 °C overnight with primary antibodies at a 1:1000 dilution, with the exception of antibodies purchased from Santa Cruz, which were used at 1:500, and GAPDH, which was used at 1:10000. The appropriate horseradish peroxidase-conjugated secondary antibodies were then added. Immunoreactive protein bands were visualized on X-ray film with an enhanced chemiluminescence detection system (GE Healthcare). Densitometric quantification of the immunoreactive bands was performed using Image J 1.29 software (NIH, Bethesda, MD). Except for Western blot for nuclear β-catenin in ILK silencing study (n = 2 from pooled 3 donors), each experiment has been completed with triple independent tests.

### Statistics

All data are presented as the mean ± S.D. calculated for each group, and at least three independent experiments were performed. The data were compared using Wilcoxon Rank-Sum Test for paired samples. SPSS 12.0 software (SPSS Inc., Chicago, IL, USA) was used for statistical analyses. Test results are reported as two-tailed *p* values, where *p* < 0.05* was considered statistically significant.

## Additional Information

**How to cite this article**: Hsueh, Y.-J. *et al.* Preservation of epithelial progenitor cells from collagenase-digested oral mucosa during *ex vivo* cultivation. *Sci. Rep.* **6**, 36266; doi: 10.1038/srep36266 (2016).

**Publisher’s note:** Springer Nature remains neutral with regard to jurisdictional claims in published maps and institutional affiliations.

## Supplementary Material

Supplementary Information

## Figures and Tables

**Table 1:** TaqMan array analysis for 92 genes associated with ILK signaling expressed by human OMECs isolated by dispase II/trypsin (D/T) or collagenase (Col.).

ΔCt		Fold change	Gene symbol	Gene Description
Col.	D/T			
10.446	7.070	10.38	ITGB2	CD18, integrin β2
7.158	3.993	8.97	MYC	c-Myc
4.992	2.168	7.08	CDH1	CD324, E-cadherin
5.380	2.724	6.30	JUN	c-Jun
4.891	2.505	5.23	AKT1	Akt/PKB
3.325	1.081	4.74	CCND1	cyclin D1
5.494	3.544	3.86	EGFR	CD20, epidermal growth factor receptor
2.308	0.377	3.81	RHOA	small GTPase protein
7.044	5.264	3.43	RAC1	rho family, small GTP binding protein
5.915	4.362	2.93	ILK	integrin linked kinase
9.157	7.970	2.28	TCF7L2	also known as TCF4
5.074	3.949	2.18	GSK3B	glycogen synthase kinase 3 beta

The table lists 12 genes with an increase in expression larger than two folds.

## References

[b1] Nakamura T. *et al.* The successful culture and autologous transplantation of rabbit oral mucosal epithelial cells on amniotic membrane. Invest. Ophthalmol. Vis. Sci. 44, 106–116 (2003). 12506062 10.1167/iovs.02-0195

[b2] Chen H. C. *et al.* Persistence of transplanted oral mucosal epithelial cells in human cornea. Invest Ophthalmol Vis Sci 50, 4660–4668 (2009). 19458337 10.1167/iovs.09-3377

[b3] Ma D. H. *et al.* Transplantation of cultivated oral mucosal epithelial cells for severe corneal burn. Eye (Lond) 23, 1442–1450 (2009). 19373264 10.1038/eye.2009.60

[b4] Amemiya T., Nakamura T., Yamamoto T., Kinoshita S. & Kanamura N. Autologous transplantation of oral mucosal epithelial cell sheets cultured on an amniotic membrane substrate for intraoral mucosal defects. PLoS One 10, e0125391 (2015). 25915046 10.1371/journal.pone.0125391 PMC4410995

[b5] Takagi R. *et al.* Cell sheet technology for regeneration of esophageal mucosa. World J. Gastroenterol. 18, 5145–5150 (2012). 23066307 10.3748/wjg.v18.i37.5145 PMC3468845

[b6] Nakamura T. *et al.* Transplantation of cultivated autologous oral mucosal epithelial cells in patients with severe ocular surface disorders. Br. J. Ophthalmol. 88, 1280–1284 (2004). 15377551 10.1136/bjo.2003.038497 PMC1772364

[b7] Nishida K. *et al.* Corneal reconstruction with tissue-engineered cell sheets composed of autologous oral mucosal epithelium. N Engl. J. Med. 351, 1187–1196 (2004). 15371576 10.1056/NEJMoa040455

[b8] Chen H. C. *et al.* Persistence of transplanted oral mucosal epithelial cells in human cornea. Invest. Ophthalmol. Vis. Sci. 50, 4660–4668 (2009). 19458337 10.1167/iovs.09-3377

[b9] Pellegrini G., Rama P., Di Rocco A., Panaras A. & De Luca M. Concise review: hurdles in a successful example of limbal stem cell-based regenerative medicine. Stem Cells 32, 26–34 (2014). 24038592 10.1002/stem.1517

[b10] De Luca M., Pellegrini G. & Green H. Regeneration of squamous epithelia from stem cells of cultured grafts. Regen Med 1, 45–57 (2006). 17465819 10.2217/17460751.1.1.45

[b11] Rama P. *et al.* Limbal stem-cell therapy and long-term corneal regeneration. N Engl J Med 363, 147–155 (2010). 20573916 10.1056/NEJMoa0905955

[b12] Pellegrini G. *et al.* The control of epidermal stem cells (holoclones) in the treatment of massive full-thickness burns with autologous keratinocytes cultured on fibrin. Transplantation 68, 868–879 (1999). 10515389 10.1097/00007890-199909270-00021

[b13] Pellegrini G. *et al.* Long-term restoration of damaged corneal surfaces with autologous cultivated corneal epithelium. Lancet 349, 990–993 (1997). 9100626 10.1016/S0140-6736(96)11188-0

[b14] Ang L. P. *et al.* Autologous serum-derived cultivated oral epithelial transplants for severe ocular surface disease. Arch. Ophthalmol. 124, 1543–1551 (2006). 17102000 10.1001/archopht.124.11.1543

[b15] Yokoo S., Yamagami S., Usui T., Amano S. & Araie M. Human corneal epithelial equivalents for ocular surface reconstruction in a complete serum-free culture system without unknown factors. Invest. Ophthalmol. Vis. Sci. 49, 2438–2443 (2008). 18515584 10.1167/iovs.06-1448

[b16] Hirayama M., Satake Y., Higa K., Yamaguchi T. & Shimazaki J. Transplantation of cultivated oral mucosal epithelium prepared in fibrin-coated culture dishes. Invest Ophthalmol Vis Sci 53, 1602–1609 (2012). 22323487 10.1167/iovs.11-7847

[b17] Hyun D. W. *et al.* Characterization of biomaterial-free cell sheets cultured from human oral mucosal epithelial cells. J Tissue Eng Regen Med (2014). 10.1002/term.1971 25407749

[b18] Dua H. S., Gomes J. A., King A. J. & Maharajan V. S. The amniotic membrane in ophthalmology. Surv. Ophthalmol. 49, 51–77 (2004). 14711440 10.1016/j.survophthal.2003.10.004

[b19] Tseng S. C. Amniotic membrane transplantation for ocular surface reconstruction. Biosci. Rep. 21, 481–489 (2001). 11900323 10.1023/a:1017995810755

[b20] Wang D. Y., Hsueh Y. J., Yang V. C. & Chen J. K. Propagation and phenotypic preservation of rabbit limbal epithelial cells on amniotic membrane. Invest. Ophthalmol. Vis. Sci. 44, 4698–4704 (2003). 14578389 10.1167/iovs.03-0272

[b21] Chen S. Y. *et al.* HC-HA/PTX3 Purified From Amniotic Membrane Promotes BMP Signaling in Limbal Niche Cells to Maintain Quiescence of Limbal Epithelial Progenitor/Stem Cells. Stem Cells 33, 3341–3355 (2015). 26148958 10.1002/stem.2091

[b22] Chen S. Y., Hayashida Y., Chen M. Y., Xie H. T. & Tseng S. C. A new isolation method of human limbal progenitor cells by maintaining close association with their niche cells. Tissue Eng. Part C Methods 17, 537–548 (2011). 21175372 10.1089/ten.tec.2010.0609 PMC3129703

[b23] Li W. *et al.* A novel method of isolation, preservation, and expansion of human corneal endothelial cells. Invest. Ophthalmol. Vis. Sci. 48, 614–620 (2007). 17251457 10.1167/iovs.06-1126 PMC3196988

[b24] Zhu Y. T. *et al.* Characterization and comparison of intercellular adherent junctions expressed by human corneal endothelial cells *in vivo* and *in vitro*. Invest. Ophthalmol. Vis. Sci. 49, 3879–3886 (2008). 18502989 10.1167/iovs.08-1693 PMC2566851

[b25] Symington B. E. Fibronectin receptor modulates cyclin-dependent kinase activity. J. Biol. Chem. 267, 25744–25747 (1992). 1464591

[b26] Mortarini R., Gismondi A., Santoni A., Parmiani G. & Anichini A. Role of the alpha 5 beta 1 integrin receptor in the proliferative response of quiescent human melanoma cells to fibronectin. Cancer Res. 52, 4499–4506 (1992). 1386557

[b27] Park J. H., Ryu J. M. & Han H. J. Involvement of caveolin-1 in fibronectin-induced mouse embryonic stem cell proliferation: role of FAK, RhoA, PI3K/Akt, and ERK 1/2 pathways. J. Cell Physiol. 226, 267–275 (2011). 20658539 10.1002/jcp.22338

[b28] Cordes N. & van Beuningen D. Cell adhesion to the extracellular matrix protein fibronectin modulates radiation-dependent G2 phase arrest involving integrin-linked kinase (ILK) and glycogen synthase kinase-3beta (GSK-3beta) *in vitro*. Br. J. Cancer. 88, 1470–1479 (2003). 12778079 10.1038/sj.bjc.6600912 PMC2741045

[b29] Chen H. M., Lin Y. H., Cheng Y. M., Wing L. Y. & Tsai S. J. Overexpression of integrin-beta1 in leiomyoma promotes cell spreading and proliferation. J. Clin. Endocrinol. Metab. 98, E837–E846 (2013). 23482612 10.1210/jc.2012-3647

[b30] Schiller H. B. *et al.* beta1- and alphav-class integrins cooperate to regulate myosin II during rigidity sensing of fibronectin-based microenvironments. Nat. Cell Biol. 15, 625–636 (2013). 23708002 10.1038/ncb2747

[b31] Chen J., Guerriero E., Lathrop K. & SundarRaj N. Rho/ROCK signaling in regulation of corneal epithelial cell cycle progression. Invest. Ophthalmol. Vis. Sci. 49, 175–183 (2008). 18172090 10.1167/iovs.07-0488

[b32] Nakamura T., Endo K. & Kinoshita S. Identification of human oral keratinocyte stem/progenitor cells by neurotrophin receptor p75 and the role of neurotrophin/p75 signaling. Stem Cells 25, 628–638 (2007). 17110619 10.1634/stemcells.2006-0494

[b33] Pellegrini G. *et al.* p63 identifies keratinocyte stem cells. Proc. Natl. Acad. Sci. USA 98, 3156–3161 (2001). 11248048 10.1073/pnas.061032098 PMC30623

[b34] Bamberger C., Hafner A., Schmale H. & Werner S. Expression of different p63 variants in healing skin wounds suggests a role of p63 in reepithelialization and muscle repair. Wound Repair Regen. 13, 41–50 (2005). 15659035 10.1111/j.1067-1927.2005.130106.x

[b35] Kang Y. A. *et al.* Copper-GHK increases integrin expression and p63 positivity by keratinocytes. Arch. Dermatol. Res. 301, 301–306 (2009). 19319546 10.1007/s00403-009-0942-x

[b36] Ma D. H. *et al.* Preservation of human limbal epithelial progenitor cells on carbodiimide cross-linked amniotic membrane via integrin-linked kinase-mediated Wnt activation. Acta Biomater. 31, 144–155 (2016). 26612415 10.1016/j.actbio.2015.11.042

[b37] Chu W. K., Dai P. M., Li H. L. & Chen J. K. Glycogen synthase kinase-3beta regulates DeltaNp63 gene transcription through the beta-catenin signaling pathway. J. Cell. Biochem. 105, 447–453 (2008). 18615589 10.1002/jcb.21839

[b38] Tetsu O. & McCormick F. Beta-catenin regulates expression of cyclin D1 in colon carcinoma cells. Nature 398, 422–426 (1999). 10201372 10.1038/18884

[b39] Hsueh Y. J. *et al.* STAT3 regulates the proliferation and differentiation of rabbit limbal epithelial cells via a DeltaNp63-dependent mechanism. Invest. Ophthalmol. Vis. Sci. 52, 4685–4693 (2011). 21447682 10.1167/iovs.10-6103

[b40] Hsueh Y. J., Kuo P. C. & Chen J. K. Transcriptional regulators of the DeltaNp63: their role in limbal epithelial cell proliferation. J. Cell. Physiol. 228, 536–546 (2013). 22806179 10.1002/jcp.24160

[b41] Chen S. Y., Mahabole M. & Tseng S. C. Optimization of *ex vivo* expansion of limbal epithelial progenitors by maintaining native niche cells on denuded amniotic membrane. Transl. Vis. Sci. Technol. 2, 1 (2013). 10.1167/tvst.2.7.1 PMC3823523 24222891

[b42] Xie H. T., Chen S. Y., Li G. G. & Tseng S. C. Limbal epithelial stem/progenitor cells attract stromal niche cells by SDF-1/CXCR4 signaling to prevent differentiation. Stem Cells 29, 1874–1885 (2011). 21948620 10.1002/stem.743

[b43] Xie H. T., Chen S. Y., Li G. G. & Tseng S. C. Isolation and expansion of human limbal stromal niche cells. Invest. Ophthalmol. Vis. Sci. 53, 279–286 (2012). 22167096 10.1167/iovs.11-8441 PMC3292364

[b44] Omoto M. *et al.* The use of human mesenchymal stem cell-derived feeder cells for the cultivation of transplantable epithelial sheets. Invest Ophthalmol Vis Sci 50, 2109–2115 (2009). 19136703 10.1167/iovs.08-2262

[b45] Oie Y. *et al.* A novel method of culturing human oral mucosal epithelial cell sheet using post-mitotic human dermal fibroblast feeder cells and modified keratinocyte culture medium for ocular surface reconstruction. Br J Ophthalmol 94, 1244–1250 (2010). 20538654 10.1136/bjo.2009.175042

[b46] Okazaki M., Yoshimura K., Suzuki Y. & Harii K. Effects of subepithelial fibroblasts on epithelial differentiation in human skin and oral mucosa: heterotypically recombined organotypic culture model. Plast. Reconstr. Surg. 112, 784–792 (2003). 12960859 10.1097/01.PRS.0000069710.48139.4E

[b47] Han B., Chen S. Y., Zhu Y. T. & Tseng S. C. Integration of BMP/Wnt signaling to control clonal growth of limbal epithelial progenitor cells by niche cells. Stem Cell Res. 12, 562–573 (2014). 24530980 10.1016/j.scr.2014.01.003 PMC3952206

[b48] Tseng S. C., Prabhasawat P., Barton K., Gray T. & Meller D. Amniotic membrane transplantation with or without limbal allografts for corneal surface reconstruction in patients with limbal stem cell deficiency. Arch. Ophthalmol. 116, 431–441 (1998). 9565039 10.1001/archopht.116.4.431

[b49] Meller D. *et al.* Amniotic membrane transplantation for acute chemical or thermal burns. Ophthalmology 107, 980-989; discussion 990 (2000). 10.1016/s0161-6420(00)00024-5 10811094

