# Supplementary material for: Preservation of epithelial progenitor cells from collagenase-digested oral mucosa during ex vivo cultivation
Source: Sci Rep. 2016 Nov 8;6:36266. doi: 10.1038/srep36266 (PMC5099970; doi:10.1038/srep36266)
Supplement: Supplementary Information [file srep36266-s1.pdf]

Supplementary Information

Preservation of epithelial progenitor cells from collagenase-digested oral mucosa during ex vivo cultivation

Yi-Jen Hsueh[1,2], Shiang-Fu Huang[3,4], Jui-Yang Lai[1,2,5,6], Shih-Chieh Ma[1,2], Hung-Chi Chen*[1,2,4], Sung-En Wu[1,2], Tze-Kai Wang[1,2], Chi-Chin Sun[7,8], Kevin Sheng-Kai Ma[9], Jan-Kan Chen[2,10], Chyong-Huey Lai[11], David Hui-Kang Ma*[1,2,8]

[1] Limbal Stem Cell Laboratory, Department of Ophthalmology, Chang Gung Memorial Hospital, Linkou, Taiwan.
[2] Center for Tissue Engineering, Chang Gung Memorial Hospital, Linkou, Taiwan.
[3] Department of Otolaryngology, Chang Gung Memorial Hospital, Linkou, Taiwan.
[4] Department of Medicine, College of Medicine, Chang Gung University, Taoyuan, Taiwan.
[5] Institute of Biochemical and Biomedical Engineering, Chang Gung University, Taoyuan, Taiwan.
[6] Biomedical Engineering Research Center, Chang Gung University, Taoyuan, Taiwan.
[7] Department of Ophthalmology, Chang Gung Memorial Hospital, Keelung, Taiwan.
[8] Department of Chinese Medicine, College of Medicine, Chang Gung University, Taoyuan, Taiwan.
[9] Department of Dentistry, College of Oral Medicine, Chung Shan Medical University, Taichung, Taiwan.
[10] Department of Physiology, College of Medicine, Chang Gung University, Taoyuan, Taiwan.
[11] Department of Obstetrics and Gynecology, Chang Gung Memorial Hospital, Linkou, Taiwan.

***Corresponding Authors:**
David Hui-Kang Ma, MD, PhD
E-mail: davidhkma@yahoo.com
Hung-Chi Chen, MD, PhD
E-mail: mr3756@cgmh.org.tw

This PDF file includes:
Figure S1, Figure S2 and Table S1

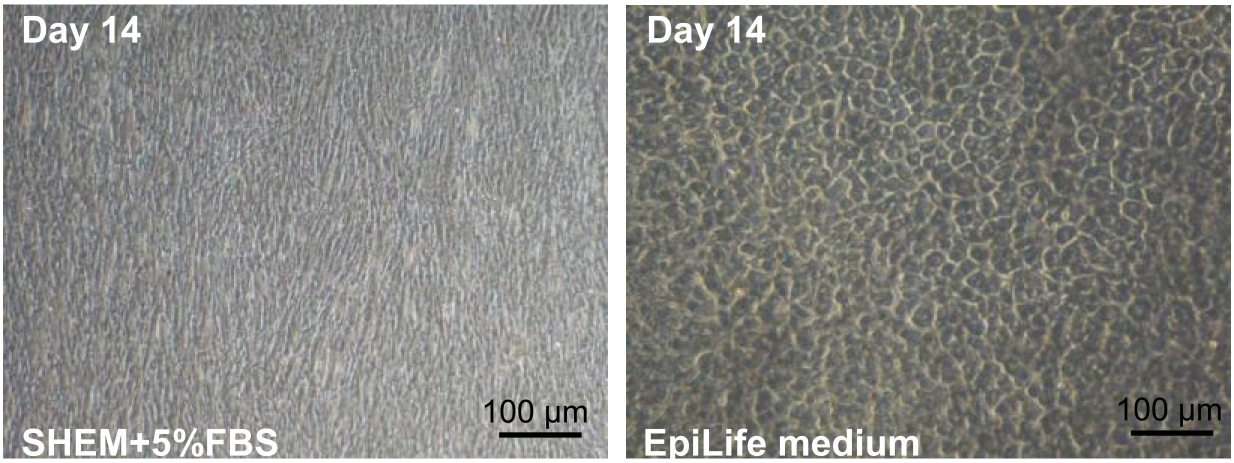

Supplementary Figure S1

Submucosal mesenchymal cells contamination in collagenase-isolated oral mucosal epithelial cells (OMECs)

Left: Culture with SHEM medium containing 5% FBS, submucosal mesenchymal cells overgrowth is often encountered in OMECs isolated by collagenase. Right: In contrast, submucosal mesenchymal cells overgrowth is suppressed when the medium is changed to serum free EpiLife medium.

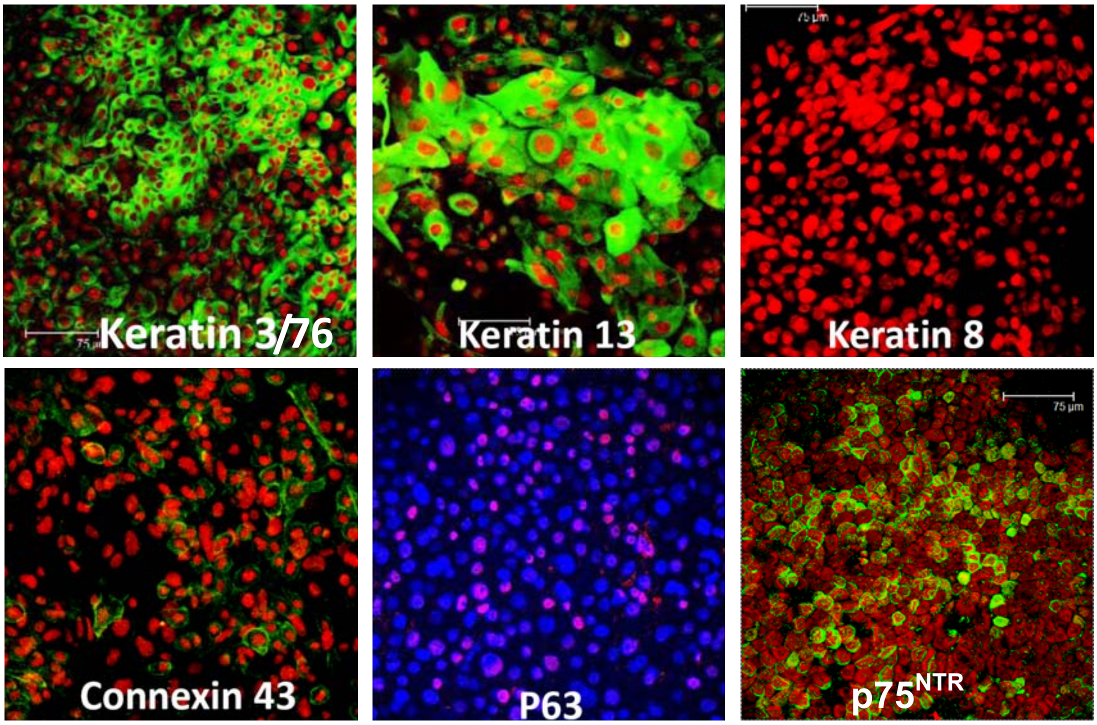

Supplementary Figure S2

Characterization of human oral mucosal epithelial cells (OMECs) isolated by collagenase

Immunoconfocal microscopy showed that, similar to normal oral mucosal epithelium, OMECs isolated by collagenase and grown on de-epithelialized AM were positive for cytokeratin 3/76, 13, and connexin 43 (green), but negative for cytokeratin 8. Prominent staining for stem cell markers p63 (red) and $p75^{NTR}$ (green) was also evident. Cell nuclei counter-stained by PI (red) or Hoechst 33342 (blue). The bars represent 75 μm.

No.	Age	Sex	Primary disease	Excision site	Microscopic finding
**IRB no: 101-0108B**					
**1**	63	M	Left recurrent buccal cancer, cT2N0M0	Left buccal	Sections show no tumor
**2**	57	M	Left buccal cancer, cT2N0M0	Left post. buccal	Sections show no tumor
**3**	53	M	Left buccal SCC, pT4aN2bM0	Left buccal	Sections show no tumor
**4**	59	M	Recurrent lip cancer, pT2N2bM0	Upper lip	Sections show no tumor
**5**	47	M	Left buccal cancer recurrence	Left buccal	Sections show no tumor
**6**	73	F	Right buccal cancer, pT3N0	Right upper buccal	Sections show no tumor
**7**	58	M	Mouth floor cancer, cT1~2N0M0	Mouth floor	No definite malignancy
**8**	42	F	Left buccal cancer, pT3N0M0	Left upper buccal	Sections show no tumor
**9**	37	M	Right buccal cancer, pT2N2b	Right buccal	Sections show no tumor
**10**	50	M	Left buccal-mouth floor cancer, pT4aN1M0	Left buccal	Sections show no tumor
**11**	45	M	Left buccal cancer,cT4aN2bM0	Left mouth floor	All margins are negative for malignancy
**12**	53	M	Left retromolar squamous cell carcinoma, cT2N0M0	Left retromolar	All margins are negative for malignancy
**13**	56	M	Left lower lip cancer, cT2N0M0	Left lower lip	Sections show no tumor.
**IRB no: 102-5895B**					
**14**	85	M	Left buccal cancer, SCC cT1N0M0 pT2N0M0	Left buccal	All margins are negative for malignancy
**15**	37	M	Right lower gum cancer, pT4aN2b	Right buccal	All margins are negative for malignancy
**16**	59	M	Right buccal squamous cell carcinoma, cT4N0M0	Right buccal	No definite malignancy is seen.
**17**	44	M	Right retromolar trigone MDSqCC, rpT2	Right retromolar trigone	Sections show no tumor
**18**	61	M	Right gum cancer, pT4aN0	Right palate	Sections show no tumor
**19**	35	M	Right maxillary sinus cancer, cT4aN2bM0	Right maxillary sinus	Sections show no tumor
**20**	54	M	Lower lip cancer	Lower lip	Sections show no tumor
**21**	66	F	Left buccal cancer, cT4bN1M0	Left buccal mucosa	Sections show no tumor
**22**	58	M	Right buccal cancer recurrence, rcT2N1M0	Right buccal	Sections show no tumor
**23**	74	M	Right buccal cancer, cT2N0M0	Buccal mucosa	Sections show no tumor
**24**	21	F	Chondroblastic osteosarcoma of Right mandible, cT2N0M0	Right mandible	Sections show no tumor
**25**	69	M	Lower lip cancer, cT2N0M	Lower lip cancer	Sections show no tumor
**26**	43	M	Right buccal cancer T1N2bM0	Right buccal	Sections show no tumor
**27**	51	M	Right buccal cancer T1N0M0	Right buccal	Sections show no tumor
**28**	37	M	Left buccal cancer, cT4aN2bM0,	Left buccal	No evidence of malignancy is seen.
**29**	51	M	Left buccal cancer, cT1N1M0, pT2N2b	Left buccal	Sections show no tumor
**30**	60	M	Right lower gum, rcT2N0, pT4aN0	Right lower gum	No evidence of malignancy is seen.

Supplementary Table S1

Demographic information of oral mucosa donors:
